# Public Perception Toward Ministry of Health Regulations for Antibiotic Dispensing and Its Impact on Pharmacy and Family Physician Visits

**DOI:** 10.7759/cureus.14981

**Published:** 2021-05-12

**Authors:** Salman AlOtieschan, Abdulrahman Alsalim, Shahad Albabtain, Munthir Almujahid, Dana Obeid, Faisal Alhabradi, Thamer Alenazi

**Affiliations:** 1 College of Medicine, King Saud Bin Abdulaziz University for Health Sciences, Riyadh, SAU; 2 College of Medicine, King Abdullah International Medical Research Center, Riyadh, SAU; 3 Department of Radiology, King Faisal Specialist Hospital and Research Centre, Riyadh, SAU; 4 College of Pharmacy, King Saud Bin Abdulaziz University for Health Sciences, Riyadh, SAU; 5 Department of Pediatrics, King Fahad Medical City, Riyadh, SAU; 6 Department of Orthopedics, King Abdulaziz Medical City, Riyadh, SAU; 7 Department of Academic Affairs, Prince Sultan Military Medical City, Riyadh, SAU; 8 Department of Medicine, King Abdulaziz Medical City, Riyadh, SAU

**Keywords:** antimicrobial resistance, over the counter antibiotics, family physician (fp), antibiotics, public perception, public health, pharmacy

## Abstract

Introduction

Antibiotic dispensing regulations have been implemented in most countries worldwide. Saudi Arabia’s Ministry of Health (MOH) recently implemented a policy for dispensing antibiotics, in which pharmacies in Saudi Arabia are strictly prohibited from dispensing antibiotics without a medical prescription. Failure to adhere to the regulations will result in a fine and may lead to the revoking of the pharmacy's license. The aim of this study was to investigate public perception among the Saudi population toward the recently implemented policy, to examine the effect of antibiotic dispensing regulations on consumers’ request for non-prescribed antibiotics at retail pharmacies, and to elucidate the implications of the recent policies on patient visitation to family physicians.

Method

A cross-sectional study was conducted using three questionnaires targeting the general public in Saudi Arabia in addition to pharmacists and family physicians working in Saudi Arabia.

Results

A total of 380 participants completed the questionnaire for the general public, 299 for the pharmacist questionnaire, and 94 for the family physician questionnaire. Most participants in the general public questionnaire obtained their antibiotics using a prescription after consulting with a family physician (72.4%). Most of these individuals also agreed with the strict regulations for antibiotic dispensing (82.1%). Most pharmacist participants either always or frequently received requests to dispense antibiotics without a prescription from customers (71.5%). In addition, most physicians (59.6%) acknowledged that patient demand increased after the implementation of the new policies.

Conclusion

Our assessment of public perception towards the recent regulation for dispensing antibiotics indicates that most participants who participated in the General Public questionnaire supported the MOH’s strict regulations for antibiotic dispensing. However, customer requests for antibiotics without a prescription from pharmacists were a bit high, which could be attributed to customers' lack of awareness of the regulations. The results also indicate an increase in the number of family physician visits after the regulations were implemented.

## Introduction

In the past few decades, dispensing of non-prescribed antibiotics has been a growing concern for the World Health Organization (WHO), especially with the dramatic increase in trajectory seen in developing countries [1]. The WHO predicts that Antimicrobial Resistance (AMR) will reach an alarming level by 2050, which might potentially trigger a pandemic [1-2]. Few countries have implemented regulations for selling antibiotics Over the Counter (OTC) [1-2]. Most of these countries were once part of the former Soviet Union [1-2]. However, most developing countries either lack strict regulatory mechanisms or their current regulations are insufficiently enforced [1-2]. Kalungia et al. investigated the prevalence of dispensing non-prescribed antibiotics by pharmacists in Zambia, reporting that all participants admitted to dispensing antibiotics without a prescription to some patients [2].

Hu and Wang surveyed Chinese immigrants residing in Australia on the question of whether they had used antibiotics in the past 12 months, and if the antibiotic was prescribed by a General Practitioner (GP) [3]. Out of the 426 participants, 170 had used antibiotics in the past year [3]. To the researchers’ surprise, half of the participants were taking antibiotics without consulting with a GP [3]. They concluded that participants with a positive GP visit experience were more likely to obtain a prescription [3].

In a study that sought to evaluate antibiotic use among adults (n = 323) in Greek urban areas, researchers found that 163 of the surveyed participants used non-prescribed antibiotics [4]. Thirty-four of these individuals were caregivers who administered courses of antibiotics to their children without a prior GP consultation [4]. It is worth noting that antibiotics in Greece are sold as OTC drugs, which might account for the high prevalence of non-prescribed antibiotics [4].

In the United States, for instance, strict regulatory policies have been implemented for dispensing antibiotics in most states, but the use of antibiotics without a prescription has increased, especially among Latinos [5]. In a study conducted in a Latino community in South Carolina, 30.6% of participants believed that antibiotics should be dispensed without a prescription [5]. Another study indicated that self-medication with antibiotics is a common practice among Hispanic households in New York [6]. Much of the problem in the rise in AMR globally lies in developing countries, where they either lack policies against dispensing non-prescribed antibiotics or they simply do not exist [6]. In certain developing countries such as Brazil, Sudan, and Bangladesh, the elevated prevalence of non-prescribed antibiotics has been associated with the growth of AMR [6].

In Saudi Arabia, several studies have been conducted over the past few years to assess public perception and awareness toward indications for antibiotic use and their associated complications [7-8]. In a survey of 473 individuals, 92% admitted to using non-prescribed antibiotics after pharmacist counseling [7]. Another study that surveyed 327 pharmacists found that 77.6% dispensed antibiotics without prescription, and dispensation occurred without the patient’s request in 95% of cases [8]. These alarming numbers and behaviors surrounding antibiotic dispensing at the level of both pharmacists and individuals in Saudi Arabia led legislators to act fast to enforce regulatory policies [7-8]. In April 2018, the Saudi Ministry of Health (MOH) enacted and began to enforce regulations for dispensing antibiotics [9].

The aim of this study is to investigate public perception among the Saudi population towards the recently implemented policies for dispensing antibiotics, to examine the effect of antibiotic dispensing regulations on consumers’ request for non-prescribed antibiotics at retail pharmacies, and to elucidate the implications of the recent policies on patient visitation to family physicians.

## Materials and methods

Study design

A cross-sectional study was done in Saudi Arabia between November 2019 and May 2020 using three self-administered questionnaires. The questionnaires used in this study were obtained from previously published articles with their permission [2,7,10].

Questionnaires

General Public

The questionnaires were self-administered by members of the general public. Questionnaires were distributed randomly in the waiting area of the National Guard Hospital in Riyadh, as well as public places in the city (mostly coffee shops and malls). All Saudi citizens above 18 years old were eligible to participate, and all non-Saudi participants or individuals younger than 18 were excluded. Participants were randomly selected regardless of their age, gender, or level of education.

Pharmacist

Google Forms was used to create the pharmacist questionnaire, which were randomly distributed electronically to all pharmacists working in two well-known pharmacy chains in Saudi Arabia. We included all Saudi and non-Saudi pharmacists working in private pharmacies in Saudi Arabia.

Family Physician

Google Forms was used to create the family physician questionnaire. We contacted the Saudi Commission for Health Specialties (SCFHS) to send it by email to all Saudi and non-Saudi physicians who are registered and working in the primary health clinics in Saudi Arabia as Family Medicine Physicians.

Data analysis

Data were checked for completeness and correctness. Categorical variables were presented as frequencies and percentages. Continuous variables were presented as mean ± standard deviation (SD). The Chi-square test was used to test the association between categorical variables, while the Mann-Whitney U test was used to examine the relationship between categorical and continuous variables. The analysis was performed with a 95% confidence interval using the Statistical Package for the Social Sciences (SPSS) version 23.0 (IBM Corp., Armonk, NY, USA).

Ethical considerations

The study was reviewed and approved by the Institutional Review Board (IRB) at King Abdullah International Medical Research Center (KAIMRC), the Ministry of National Guard Health Affairs (MNGHA), Riyadh, Saudi Arabia. The study’s protocol number is #RC19/079/R. Participation was voluntary and informed consent was obtained from all participants prior to administering the questionnaire. All collected data were obtained confidentially, kept confidential, and stored in a secure place.

## Results

General public

Of the 400 questionnaires distributed to the general public, 380 were completed and returned, which led to a response rate of 95%. For the remaining 20 questionnaires, either the participants refused to participate, or the data were incomplete. The mean age of the participants was 34.01 ± 12.84 years, and 58% were female. More than half (56.1%) held a bachelor’s degree (Table 1).

**Table 1:** Socio-demographic characteristics of the general public participants (n = 380)

Characteristics	N (%)
Age in years	
Mean ± SD	34.01 ± 12.84
Gender	
Male	160 (42.1)
Female	220 (57.9)
Level of education	
Uneducated/illiterate	17 (4.5)
Secondary school (high school)	77 (20.3)
Undergraduate (bachelor's)	213 (56.1)
Graduate (PhD, MSc, etc.)	50 (13.2)
Diploma	23 (6.1)

Most participants obtained antibiotics with a prescription after consulting with a family physician (72.4%). Only a few participants obtained antibiotics after consulting with a pharmacist with a prior valid prescription (6.8%), and the remainder obtained their antibiotics without a prescription (4.7%). In addition, a handful of participants suggested that they would stop their antibiotic regimen as soon as they felt better (28.7%), while most were committed to finishing their course (71.3%). Surprisingly, almost one-quarter of the participants were willing to share their antibiotics with others (23.7%) (Table 2).

**Table 2:** General public's responses to the questionnaire

Questions	Answers	N	%
How do you usually get your antibiotics?	Prescription after consulting a Family Physician	275	72.4
	Prescription after consulting a Pharmacist	26	6.8
	Without a prescription	18	4.7
	Sometimes with prescription and sometimes without	61	16.1
If your Family Physician prescribed you an antibiotic, and you think you do not need it, what would you do?	I will use the antibiotics anyways.	92	24.2
	I will tell my Family Physician I do not want to take it.	166	43.7
	I will take the antibiotic but will not use it.	61	16.1
	I will consult another physician.	61	16.1
If you got better before finishing your antibiotics course, what would you do?	I will continue taking the antibiotic until I finish the course.	271	71.3
	I will stop taking the antibiotic.	109	28.7
If you already have an antibiotic, would you be willing to share it with others?	No	290	76.3
	Yes	90	23.7
Did you hear that it is illegal to prescribe antibiotics without a prescription?	No	38	10.0
	Yes	342	90.0
Where did you hear that it is illegal to prescribe antibiotics without a prescription?	Did not hear about it	38	10.0
	Physician/Hospital	73	19.2
	Pharmacist/Pharmacy	90	23.7
	Ministry of Health Advertisements	81	21.3
	News/Social media	67	17.6
	Friends/Family	31	8.2
Do you agree with strict regulation against dispensing non-prescribed antibiotics?	Strongly Agree	237	62.4
	Agree	75	19.7
	Neutral	37	9.7
	Disagree	20	5.3
	Strongly Disagree	11	2.9
Do you agree with implementing penalties against dispensing non-prescribed antibiotics?	Strongly Agree	173	45.5
	Agree	85	22.4
	Neutral	71	18.7
	Disagree	29	7.6
	Strongly Disagree	22	5.8
Do you prefer buying an antibiotic without a physician's prescription with a higher price instead of going through the trouble of getting a prescription?	No	295	77.6
	Yes	85	22.4
Are you going to adhere to the new regulation against buying non-prescribed antibiotics that was implemented by the Ministry of Health?	Yes, I will strongly adhere.	194	51.1
	I will adhere	114	30.0
	Not sure	50	13.2
	I will not adhere	22	5.8

Ten percent of the respondents did not know that it was illegal to prescribe antibiotics without a prescription. Among those who knew, they mostly knew from pharmacists/pharmacies (23.7%), while MOH advertisement for illegal antibiotic dispensing without a prescription was the second most common source of participants awareness (21.3%). Most participants agreed with the strict regulations (82.1%) and penalties for dispensing non-prescribed antibiotics (67.9%). However, almost one quarter stated that they would prefer to purchase antibiotics at a higher price without a physician's prescription instead of going through the trouble of getting a prescription (22.4%). Regarding the participants’ adherence, the overwhelming majority declared they would conform to the new regulations for antibiotic dispensing implemented by the MOH (81.1%) (Table 2).

**Figure 1:**
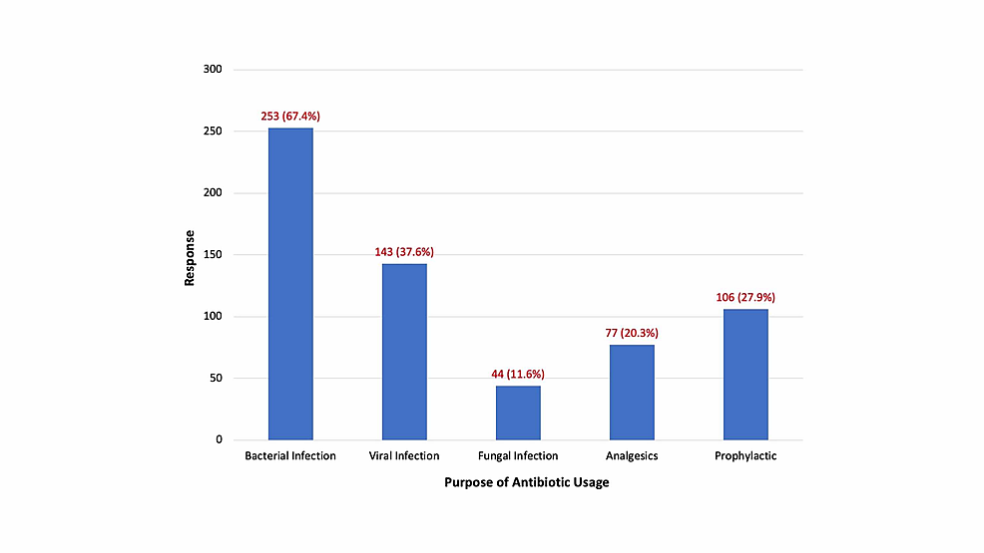
General public's responses on the purpose of antibiotic usage

Most respondents (67.4%) knew that antibiotics are prescribed to treat bacterial infections (Figure 1). However, knowledge about the harmfulness of antibiotics was not associated with any socio-demographic group (p-value > 0.05) (Table 3). Additionally, responses to all questions were statistically significant to a different gender (Table 4). Finally, no statistical correlation was identified between the participants' answers and their educational level (p-value > 0.05).

**Table 3:** Relationship between ‘are antibiotics harmful’ and baseline characteristics

Characteristics	p-value
Age in years	0.212
Gender	0.053
Region	0.307
Level of education	0.100

**Table 4:** Relationship between specific questions and gender *Odds ratio could not be statistically computed for questions with more than two variables. It was only calculated for questions with dichotomous variables (i.e., questions that had only two variables).

Specific questions	Answers	Male (%)	Female (%)	p-value	Odds ratio
Did you hear that it is illegal to prescribe antibiotics without a prescription?	No	16.9	5.0	< 0.001	3.857
	Yes	83.1	95.0		
Do you agree with strict regulation against dispensing non-prescribed antibiotics?	Strongly Agree	60.0	64.1	< 0.001	*
	Agree	12.5	25.0		
	Neutral	14.4	6.4		
	Disagree	7.5	3.6		
	Strongly Disagree	5.6	0.9		
Do you agree with implementing penalties against dispensing non-prescribed antibiotics?	Strongly Agree	42.5	47.7	< 0.001	*
	Agree	18.8	25.0		
	Neutral	16.9	20.0		
	Disagree	11.3	5.0		
	Strongly Disagree	10.6	2.3		
Do you prefer buying an antibiotic without a physician's prescription with a higher price instead of going through the trouble of getting a prescription?	No	71.3	82.3	< 0.001	0.534
	Yes	28.7	17.7		
Are you going to adhere to the new regulation against buying non-prescribed antibiotics that was implemented by the Ministry of Health?	Yes, I will strongly adhere.	46.3	54.5	< 0.011	*
	I will adhere	23.8	34.5		
	Not sure	17.5	10.0		
	I will not adhere	12.5	0.9		

Pharmacists

Pharmacists from all major areas of Saudi Arabia were included in this study. Approximately 81% of pharmacists stored their antibiotics. Most pharmacists either always or frequently received requests to dispense antibiotics without a prescription from customers (71.5%). Three-point four percent of pharmacists responded that customers could purchase antibiotics without a prescription.

Most customers requested antibiotics either because they suspected infection or because they had used it before and were unwilling to visit a hospital for medical consultation (56.8%). The overwhelming majority of pharmacists (80.3%) acknowledged that antibiotic misuse has a substantial community impact, and 59.2% believed that public and professional education was required on the rational use of antibiotics to prevent misuse. Interestingly, very few pharmacists warned customers of the potential side effects associated with using specific antibiotics (2.3%), and only slightly more educated patients on correct dosing and potential side effects (7.7%). Further questions related to pharmacists’ antibiotic dispensing behaviors are presented in (Table 5).

**Table 5:** Pharmacists' responses to the questionnaire

Questions	Answers	N	%
Customers per day	Less than 100	83	27.8
	100-200	158	52.8
	More than 200	58	19.4
Stock antibiotics in the pharmacy?	No	56	18.7
	Yes	243	81.3
Request for antibiotics without a prescription?	Rarely	85	28.4
	Frequently	132	44.1
	Always	82	27.4
Can buy without a prescription?	No	289	96.6
	Yes	10	3.4
Ask before buying?	No	9	3.0
	Yes	290	97.0
Which antibiotics do clients frequently request?	Augmentin	207	69.2
	Fusidic Acid	30	10.0
	Metronidazole (Flagyl)	8	2.7
Main reason for their requesting?	Suspected infection	88	29.4
	Used the drug before and they do not want to go to hospital and pay money	82	27.4
	Recommendation from family member/friend	18	6.0
	Other/None	57	19.1
Medicine information you tell your clients?	Dosage instructions	100	33.5
	Common side effects	7	2.3
	Dosage instructions and common side effects	23	7.7
	Other/None	115	38.5
Do you usually suggest choices of alternative antibiotics?	No	213	71.3
	Yes	32	10.7
Level of impact do you think the misuse of antibiotics in the community?	Huge impact	240	80.3
	Moderate impact	47	15.7
	Low impact	8	2.7
	No impact	4	1.3
What measures do you think should be put in place to limit misuse of antibiotics?	Public & professional education on rational use of antibiotics required	177	59.2
	Stronger enforcement of medicines regulations	75	25.1
	Other/None	47	15.7

Family physicians

A total of 94 Family Physicians participated in the study, including Consultants (37.2%), Associated/Assistant Consultants (19.1%), Residents (31.9%), and General Practitioners (11.7%). Most participants were Saudi nationals (68.1%). All participants had previously prescribed antibiotics to all age groups for various reasons.

After the MOH’s implementation of regulatory policies for antibiotic dispensing, most physicians (58%) acknowledged that between 11% and 20% of consultations resulted in antibiotics being prescribed to patients. About 27.7% of physicians acknowledged that more than 20% of visits resulted in an antibiotic prescription. Furthermore, 66% of physicians stated that they occasionally prescribe antibiotics for upper respiratory tract infections (URTIs), cold, or flu, while 30% had never prescribed antibiotics for the same reason. More than half of respondents (54.3%) did not prescribe antibiotics without a clear indication for their need, while one-third (33%) admitted to dispensing antibiotics even when not clinically indicated. More than one-third of physicians attributed their prescription of antibiotics to patients to diagnostic uncertainty. Out of the signs that might influence a physician to prescribe antibiotics, the most common sign was the presence of tonsillar exudate, where the overwhelming majority (96.8%) were very likely or likely to prescribe antibiotics (Table 6).

**Table 6:** General Physicians' responses to the questionnaire URTI: upper respiratory tract infections; MOH: Ministry of Health

Questions	Answers	N	%
Over the past one year, what was the percentage (%) of consultations (including all consultations, not limited to certain illnesses) that had led to antibiotic prescription	< 5%	9	9.6
	5-10%	4	4.3
	11-20%	55	58.5
	> 20%	26	27.7
Do you think antibiotics are useful in treating patients with URTI/cold/flu?	Yes	1	1.1
	Occasionally yes	30	31.9
	No	63	67.0
How often do you prescribe antibiotics to patients with URTIs/cold/flu?	Very often	1	1.1
	Often	1	1.1
	Sometimes	62	66.0
	Never	30	31.9
Have you prescribed antibiotics to patients with URTIs/cold/flu in cases where the prescription might not be necessary/could be optional?	Yes, often	12	12.8
	Yes, occasionally	31	33.0
	Never	51	54.3
How often do your patients / their carers request antibiotics when consulting for URTIs/cold/flu?	Always	16	17.0
	Very often	44	46.8
	Often	16	17.0
	Sometimes	17	18.1
	Never	1	1.1
Please rate the impact of patients’/their carers’ expectations on your prescription of antibiotics for URTIs/cold/flu?	Always	8	8.5
	Very often	24	25.5
	Often	29	30.9
	Sometimes	24	25.5
	Never	9	9.6
When you prescribe antibiotics, how often do you remind patients that improper use of antibiotics will increase antimicrobial resistance?	Always	55	58.5
	Very often	22	23.4
	Often	15	16.0
	Sometimes	2	2.1
How often do you advise patients on self-management (e.g., bed rest, drink plenty of fluids, hand hygiene, wear a surgical mask) when they have URTIs/cold/flu?	Always	72	76.6
	Very often	15	16.0
	Often	5	5.3
	Sometimes	2	2.1
How often do you discuss with patients that antibiotics cannot cure viral infections like URTIs/cold/flu?	Always	70	74.5
	Very often	19	20.2
	Often	3	3.2
	Sometimes	1	1.1
	Never	1	1.1
Please rate the severity of antimicrobial resistance in Saudi Arabia	2	9	9.6
	3	41	43.6
	4	36	38.3
	5	8	8.5
To tackle the problem of antimicrobial resistance in Saudi Arabia, please estimate the percentage of consultations (including all consultations, not limited to certain illnesses) that you could further reduce antibiotic prescription without harming	o Mean ± SD	17.02 ± 25.13	
Did you receive any promotional materials related to Safe Use of Antibiotics from the MOH?	No	51	54.3
	Yes	43	45.7
Did you use the promotional materials in your practice? Please select all the answers that apply (multiple answers)	Stick the poster on the clinic wall	21	22.3
	Educate patients using the pamphlets	31	33.0
	Remind patients to enhance personal hygiene while taking antibiotics using cue cards	25	26.6
Have your patients asked more often whether antibiotics are prescribed since May 2018, since enforcement of antibiotic regulation laws by the MOH?	No	42	44.7
	Yes	52	55.3
Have your patients demand antibiotics less since May 2018, since the enforcement of antibiotic regulation laws by the MOH?	No	56	59.6
	Yes	38	40.4
In your opinion, what will be the effective ways to promote the safe use of antibiotics by doctors in Saudi Arabia? Please select all the answers that apply (multiple answers).	Printed education materials.	47	50.0
	Guidelines	41	43.6
	Educational meetings	63	67.0
	Antibiotic Stewardship Program	56	59.6

Most physicians (98.2%) almost always or often mentioned to their patients the possibility of aggravating AMR if they improperly used their antibiotic regimen. Furthermore, most respondents (97.9%) advised their patients to adopt lifestyle modification and better self-management (e.g., bed rest, hydration, and mask-wearing). A majority of physicians discussed with patients the ineffectiveness of antibiotics against viral illnesses. Regarding physician perceptions of AMR severity in Saudi Arabia, most (90.4%) rated severity as 3 or more on a 5-point scale. More than half (54.3%) did not receive supplemental information from the MOH regarding the safe use of antibiotics. Finally, in terms of patient demand for antibiotics after the implementation of the MOH’s regulatory policies, most physicians (59.6%) acknowledged that demand increased (Table 6). Other antibiotic prescription behaviors are presented in (Table 6).

## Discussion

General public

The aim of this study was to examine the impact of the policies for regulating antibiotic dispensing introduced by the MOH in April 2018 in the Kingdom of Saudi Arabia. We sought to assess the impact of the new policies on the public perception of antibiotic dispensing with prescription, pharmacy visits, and changes in requests for antibiotics when visiting family physicians. In this way, we attempted to establish a cross-sectional assessment of current practices and the public stance on the regulations for antibiotic dispensing in Saudi Arabia.

The overwhelming majority of participants were friendly and curious to learn more about the effect of AMR on public health. Among the surveyed participants, most obtained their antibiotics through legal means (72.4%). In a study conducted in Saudi Arabia before the implementation of the regulatory policies by the MOH, 48% of the participants admitted to obtaining antibiotics without a prescription [7]. Clearly, the use of antibiotics with a prescription has increased since the enforcement of the regulatory policy, but the cause of this adherence is unclear. It could be due to MOH’s strict supervision or growth in public awareness or both or some other undetermined factor.

Approximately one-third of the participants indicated that they were likely to stop their antibiotic course midway through if they felt better (28.7%). This is one of the factors that has contributed to the global rise of AMR. An alarming observation was that almost one-quarter (23.7%) of the surveyed participants were willing to share their antibiotics if asked. These alarming behaviors require further investigation to identify possible factors that contribute to them. Compared to other communities, such as Chinese migrants in Australia, 3.4% indicated that they had used non-prescribed antibiotics obtained from friends [3]. A systematic review of studies from several European countries found that 81% of those who had obtained an antibiotic without a prescription had received it from a friend or relative [11]. This type of behavior is seen globally, however, in varying degrees among different communities.

The medical community in Saudi Arabia is advised to place greater emphasis on educating patients and raising awareness of the potential hazards of using antibiotics without physician supervision. Most members of the general public were likely to adhere to regulatory policies for dispensing antibiotics. Noteworthily, almost one-quarter of the participants (22.4%) were more willing to purchase antibiotics without a prescription than to book an appointment with a family physician. Similar behaviors have been identified in other communities, for instance, members of the Asian community in Australia have highlighted barriers to GP visits as the main reason for their seeking antibiotics without a prescription [3]. More than half of the participants who obtained antibiotics without a prescription attributed it to communication breakdown with the GP, the high cost of GP visits, no spare time to visit a GP, and GP trust issues [3].

In this study, public perception towards indications for antibiotic use was alarming. A handful of respondents suggested that antibiotics would help them treat the common flu and other viral illness. Some also went further and suggested the use of antibiotics as analgesics. Although the majority knew that antibiotics are primarily used to treat bacterial infections, some suggested that they can also be used to treat viral and fungal infections, or that they can serve as analgesics. Therefore, our results indicate a misconception regarding the simple indication for antibiotic usage. We recommend that futures campaigns for antibiotic awareness targeting audiences nationwide should incorporate a simplified format in order to convey the proper and effective usage of antibiotics and the potential danger of AMR.

Our results showed that the level of education was not associated with the degree of knowledge of antibiotic harmfulness. Furthermore, there was a significant relationship between gender and prior knowledge of the new regulatory policies. Gender differences in the proper/improper use of antibiotics have been observed in other communities. For instance, in Greece, it was found that males tend to adhere less to the correct dosing compared to females [4]. Additionally, older female participants and females with a high education level are associated with higher rates of non-prescribed antibiotic use [4]. This is surprising given that one would expect well-educated groups to have more knowledge of the implications of non-prescribed antibiotics, and thus greater caution [4]. Noteworthily, these results are inconsistent with the results of the present study. This could be related to several reasons, the main difference being the geographic setting. In particular, suburban areas were targeted in the other study, whereas in our study, we targeted the general public in an urban area (i.e., the capital city of Riyadh). 

Comparing our results with previous studies in Saudi Arabia regarding public attitudes toward obtaining non-prescribed antibiotics, we can clearly see a change in public perception towards antibiotics. In a 2016 study by Bin Nafisah et al., 48% of participants obtained antibiotics without a prescription [7]. In our study, we found that the rate of non-prescribed antibiotics decreased, with only 4.7% of participants obtaining antibiotics without a prescription. This significant decrease clearly shows an association with recent regulatory policies by the Saudi MOH. However, our data do not indicate whether the change in the rate of non-prescribed antibiotics is due to higher awareness in the general public or simply due to adherence to regulatory policy and fear of penalties, further research in that area is warranted.

Pharmacists

Approximately 71.5% of pharmacists reported that either always or frequently been asked by customers to dispense antibiotics, and only 3.3% of them dispensed antibiotics without prescription. This can be attributed to customers' lack of awareness of the recent regulations for dispensing antibiotics enforced by MOH. In a study conducted prior to the enforcement of regulatory policies for dispensing antibiotics in May 2018, involving a simulated patient presenting to a pharmacy, 82% of pharmacists prescribed antibiotics [12]. Two studies were conducted in Jeddah and Riyadh between late 2010 and early 2011, in which it was found that 97.9 % and 77.6% of pharmacists dispensed antibiotics without a prescription, respectively [13].

A recent study in Saudi Arabia compared the impact of the new regulations on community pharmacists, and it was conducted in two phases, pre-implementation and post-implementation of the new regulations [14]. In both phases, the study found a dramatic decrease in pharmacists’ antibiotic dispensing behavior [14]. Before the new regulations, 96.6% and 87.7% of pharmacists would have prescribed antibiotics for both pharyngitis and urinary tract infections (UTI) scenarios, whereas after the implementation it dropped to 12.1% and 5.2%, respectively [14]. Alrasheedy’s findings were similar to those of our current study, where we found that after implementing the new regulations, many community pharmacists stopped dispensing antibiotics without a prescription [14]. It is possible to attribute this behavior either to the new regulation or the penalties associated with non-adherence; further investigation is needed.

In a study that was conducted among community pharmacists working in Makkah province, they found that the main reasons for patients requesting antibiotics without prescription were patients' unwillingness to consult a physician for non-serious infection, inability to afford consultation, and pharmacist's good knowledge about antibiotic use [15]. The latter is much concerning, as there is a misconception among the community that pharmacists are eligible to prescribe antibiotics based on their symptoms without resorting to medical consultation. That could be a potential reason for most pharmacist participants either always or frequently received requests to dispense antibiotics without a prescription from customers (71.5%). Comparing Saudi Arabia to other developing countries that have established new enforcement regulations, including Chile and Mexico, Chile introduced the enforcement regulations in 1999, while Mexico introduced them in 2010 [16-17]. In both countries, a noticeable decrease has occurred in antimicrobial consumption [16-17].

Family physicians

More than half of the participating physicians noticed an increase in demand for antibiotics after the introduction of the MOH’s regulatory policies (59.6%). We could not find any local studies that assessed patient demand for antibiotics prior to the implementation of the MOH’s policies. However, in comparison, in a study done by the Department of Health in Hong Kong, only 16% of patients asked for antibiotics more often from their primary care physician after launching the community promotion campaign in March 2011 [10].

In our study, 66% of physicians prescribed antibiotics for patients presenting with signs and symptoms of cold, flu, or URTI without a clear indication of bacterial infection. This is clearly an issue to be addressed and should be further investigated. Several studies have indicated that dispensing antibiotics for non-bacterial infections is implicated in the rise of AMR [6,11]. Fortunately, most physicians do educate their patients on the negative effects of AMR if a course of antibiotics is improperly followed, which is consistent with the findings of Al-Homaidan and Barrimah [18].

Moreover, we recommend that more support should be provided by the MOH to primary care providers regarding the safe use of antibiotics. This is based on the finding that 54.3% of physicians did not receive any support materials from the MOH on safe antibiotic use. In comparison, in the study carried by the Department of Health in Hong Kong, 82% of physicians received materials related to the safe use of antibiotics [10]. In Palms et al.’s assessment of antibiotic prescription in the United States, researchers found that a substantial number of prescriptions were given without a clear indication (39%) [19]. Another earlier study demonstrated that 30% of outpatient antibiotic prescriptions were inappropriately given [20]. Compared to our result, 45.8% either often or occasionally made potentially unnecessary antibiotic prescriptions. This topic highlights the importance of further investigation into the criteria being used by family physicians nationwide in dispensing antibiotics and to evaluate for any discrepancies with current MOH guidelines for antibiotic dispensing. Finally, future studies should focus on the effects of social media platforms as means to enhance public perception regarding the proper use of antibiotics.

Limitations

The general public questionnaire was limited to a single geographical area, Riyadh city. The pharmacist questionnaire was only distributed to pharmacists working in two well-known pharmacy chains in Saudi Arabia. 

## Conclusions

This study’s assessment of public perception towards the recent regulations implemented by the MOH in the Kingdom of Saudi Arabia for dispensing antibiotics indicates that most participants of the general public, as well as pharmacists, support those measures. In addition, compared to the results of previous studies in Saudi Arabia prior to the implementation of the dispensing regulations for antibiotics, the prevalence of non-prescribed antibiotic dispensing has fallen sharply after the enforcement of the regulation. Most pharmacists reported that they either always or frequently receive requests from customers to dispense antibiotics without a prescription, which is likely attributed to customers' lack of awareness. Most participants were found to obtain their antibiotics through prescription, but the results also show an increase in the incidence of family physician visits after the enforcement of the regulation. A handful of participants were willing to share their antibiotics if asked, which is an alarming behavior that should be addressed in future research. In this study, we cannot assess whether public support for MOH regulation is due to public awareness of the consequence of improper use of antibiotics or simply due to the fines associated with obtaining non-prescribed antibiotics. Further research should examine the factors influencing public attitudes toward antibiotic usage and dispensing as well as effective awareness campaigns addressing the proper usage of antibiotics.
